# The association between three prevalent autoimmune disorders and the likelihood of developing prostate cancer: a Mendelian randomization study

**DOI:** 10.1038/s41598-024-62716-6

**Published:** 2024-05-23

**Authors:** Xiaoqian Deng, Shiwei Sun, Wei Yao, Peng Yue, Fuyu Guo, Yue Wang, Yangang Zhang

**Affiliations:** 1 Third Hospital of Shanxi Medical University, Shanxi Bethune Hospital, Shanxi Academy of Medical Sciences, Tongji Shanxi Hospital, Taiyuan, 030032 China; 2 Department of Urology, Taiyuan Central Hospital of Shanxi Medical University, Taiyuan, 030000 China; 3 Shanxi Bethune Hospital, Shanxi Academy of Medical Sciences, Tongji Shanxi Hospital, Third Hospital of Shanxi Medical University, Taiyuan, 030032 China; 4 Tongji Hospital, Tongji Medical College, Huazhong University of Science and Technology, Wuhan, 430030 China

**Keywords:** Autoimmune disorders, Prostate cancer, Rheumatoid arthritis, Systemic lupus erythematosus, Cancer risk, Genome-wide association study, Hyperthyroidism, Mendelian randomization, Cancer, Genetics, Immunology, Oncology, Urology

## Abstract

Numerous studies establish a significant correlation between autoimmune disorders (AIDs) and prostate cancer (PCa). Our Mendelian randomization (MR) analysis investigates the potential connection between rheumatoid arthritis (RA) and PCa, aiming to confirm causal links between systemic lupus erythematosus (SLE), hyperthyroidism, and PCa. Summary statistics from genome-wide association studies provided data on PCa and three AIDs. MR analysis, using IVW as the main approach, assessed causal relationships, validated by sensitivity analysis. IVW revealed a correlation between genetically anticipated RA and PCa, notably in Europeans (OR = 1.03; 95% CI 1.01–1.04, *p* = 2*10−5). Evidence supported a lower PCa risk in individuals with SLE (OR = 0.94; 95% CI 0.91–0.97, *p* = 2*10−4) and hyperthyroidism (OR = 0.02; 95% CI 0.001–0.2, *p* = 2*10−3). Weighted mode and median confirmed these findings. No pleiotropic effects were observed, and MR heterogeneity tests indicated dataset homogeneity. Our study establishes a causal link between RA, SLE, hyperthyroidism, and PCa.

## Introduction

In 112 countries, prostate cancer (PCa) is the most prevalent cancer and ranks as the fourth most common disease and the fifth leading cause of cancer-related deaths worldwide. In males, PCa is the second most common cancer, with an estimated yearly occurrence of around 1.4 million cases^1,2^. Although the cause of PCa remains complex, the known risk factors for PCa are limited to increasing age, family history of this cancer, specific genetic mutations (such as BRCA1 and BRCA2), and conditions like Lynch syndrome^2^. However, these risk factors alone do not adequately explain the variation in PCa incidence in the population^3^. Hence, it is crucial to improve the identification of high-risk factors for PCa and implement early detection and timely intervention in individuals deemed to be at high risk, aiming to decrease PCa-related morbidity and mortality^4,5^.

Autoimmune disorders, also known as AIDs, are a wide range of conditions characterised by the immune system's dysregulation, leading to abnormal responses from B and T cells towards normal components of the host^6^. These disorders can affect any organ system, impact individuals of all ages, and are present in almost 10% of the population^7,8^. They consume substantial healthcare resources, impose a heavy burden on patients, and have a significant economic influence^9^. The prevalence of various AIDs is on the rise, particularly in the cases of rheumatic diseases and hyperthyroidism (Graves' disease)^10,11^. Rheumatoid arthritis (RA) and systemic lupus erythematosus (SLE) are two commonly occurring rheumatic diseases. RA has a global prevalence of approximately 0.5% to 1%^12^, while SLE exhibits notable variations in prevalence and is widespread among North American and African ethnic groups^13^. Both diseases share a close relationship with genetic and environmental factors in their pathogenesis, leading to chronic inflammation and the production of autoantibodies that can impact multiple organs and tissues within the body. RA primarily affects the joints, while SLE impacts the skin, joints, kidneys, and other organs^14,15^. Hyperthyroidism typically manifests as an autoimmune condition where the thyroid gland excessively produces and releases thyroid hormones, leading to elevated hormone levels in the body and reduced TSH levels in the bloodstream. The global prevalence of hyperthyroidism is about 0.2–1.3 percent. One of the most common causes is Graves' disease, which accounts for 70% of morbidity^16^.

The long-term care of patients with AIDs raises significant worry regarding cancer morbidity and mortality^17^. Numerous studies indicate a close connection between these two conditions, although the precise underlying mechanisms and physiological processes remain incompletely understood^18,19^. This study specifically examines the causal relationship and potential interactions among the three prevalent AIDs and PCa. In recent, there has been proof of a causal connection between systemic lupus erythematosus (SLE), overactive thyroid, and prostate cancer (PCa). Additionally, both SLE and hyperthyroidism have been identified as protective factors against PCa, reducing the likelihood of developing it^20,21^. Due to limited research in this field, the connection between RA and a chance of PCa is still unknown. A recent study, an 8-year cohort study using a large group of US veteran men and the most extensive research on PCa in RA so far, revealed that patients with RA only had a slightly elevated risk of PCa (HR 1.12 [95% CI 1.04–1.20])^22^. Nonetheless, there are inevitably unmeasured or undetected confounding factors that may contribute to bias and do not support a cause-and-effect association between RA and PCa.

To fill this research void and investigate the possible cause-and-effect connection between RA and PCa, we performed a two-sample Mendelian randomization (MR) . This study presents fresh evidence supporting the connection between RA and PCa, while also confirming the causal relationship between SLE, hyperthyroidism, and PCa. These findings may offer insights into the correlation between autoimmune disorders and PCa, ultimately enhancing patient management, early detection, and prognosis in clinical practise.

## Methods

### Overall study design

**Figure 1:**
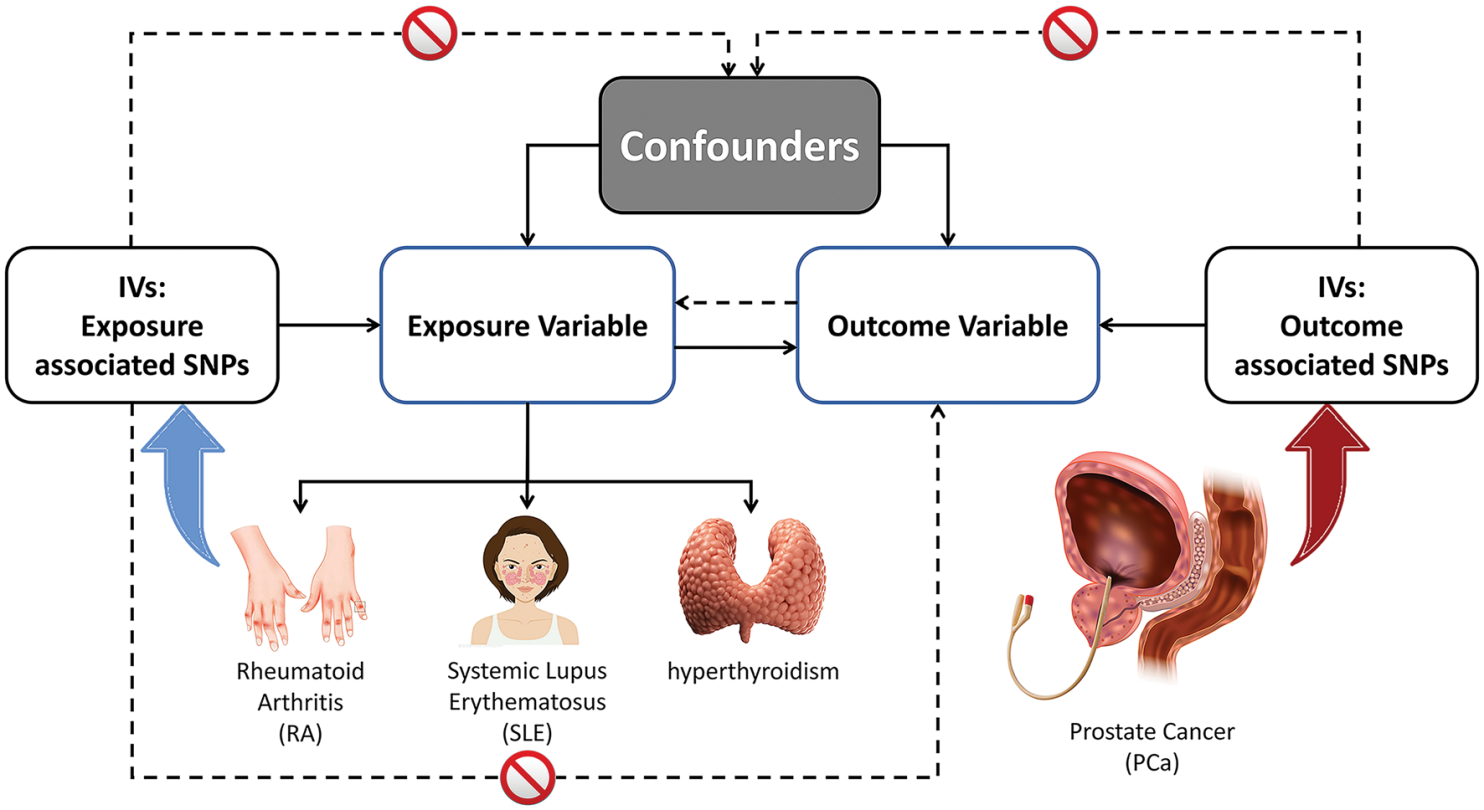
SNPs used as IVs in the two-sample MR analysis. (Single nucleotide polymorphisms (SNPs) used as IVs in the two-sample MR analysis ought to adhere to the following three fundamental presumptions: (1) IVs are strongly correlated with exposure; (2) IVs are unrelated to any confounding variables; and (3) IVs only have an impact on the outcome through exposure. The MR framework's directed graph looks into the connection between RA and AIDs).

Mendelian randomization relies on publicly available data and does not involve animal or human experiments, thus it does not require ethical approval. The data utilised in this research was obtained from published studies and publicly available databases^23^. Therefore, there was no need for additional sanctions^24^. To examine the causal relationship between AIDs and PCa, a study using the two-sample Mendelian randomization (MR) approach was conducted. The present investigation classified single nucleotide polymorphisms (SNPs) as instrumental variables (IVs). Using SNPs as a modelling technique similar to randomised controlled trials enables the discovery of causal connections between exposure traits like RA, SLE, hyperthyroidism, and outcome traits, particularly PCa (Fig. 1).

### Data sources

#### Genetic instrument variants for exposure

The exposure datasets generated in this research are available in the MR-base repository, [https://gwas.mrcieu.ac.uk/datasets/]. Specifically, “ebi-a-GCST90013534” for RA, “finn-b-SLE_STRICT” for SLE, “ukb-b-20289” for hyperthyroidism. SNPs were chosen based on comprehensive criteria. Initially, SNPs were chosen based on a strong genome-wide association with AIDs, using a significance level threshold of *P* < 5 × 10E−8. Genetic variants within close genomic regions are often co-inherited due to linkage disequilibrium (LD), where the presence of LD means that the information provided by each variant does not exist in isolation from one another. When these genetic variants are used as instrumental variables (IVs), this nonrandom association may affect the estimation of effects. To quantify and address the LD problem, we used two parameters: r^2^ and kb r^2^ measures the correlation between pairs of SNPs, with r^2^ = 1 indicating complete LD, while r^2^ = 0 indicates the presence of complete linkage equilibrium between two SNPs, reflecting a completely randomized arrangement. the kb parameter refers to the genomic distance taken into account by the LD, with closer genetic loci on chromosomes tending to be together genetically, thus increasing their r^2^ values. To ensure the independence of our genetic variants, we set strict clustering thresholds with a window size of 10,000 kb and a maximum linkage disequilibrium of r^2^ = 0.01. This threshold has been selected in many previous studies and significantly reduces the effect of LD on our selected SNPs. The SNP selection and clustering process was performed using the "TwoSampleMR" (version 0.5.6) package in the R software (version 4.2.1). Furthermore, the relationship between the instrumental variables (IVs) and the exposure factors was evaluated by analysing the F-statistic of the SNPs. Typically, IVs with an F-statistic exceeding 10 are regarded as unbiased indicators^25^.

#### Study outcome: prostate *cancer*

The outcome datasets named used in this research were acquired from the MR-base repository, [https://gwas.mrcieu.ac.uk/datasets/], “ieu-b-85”for RA, which consisted of 79,148 cases and 61,106 controls.

### Statistical analysis

To offer a credible explanation for the MR analysis, one must take into account the hypotheses mentioned^26^. (i) The connection between instrumental variables (IVs) and AIDs has been firmly established in scholarly works. (ii) PCa was exclusively influenced by the effects of IVs associated with AIDs. The IVs confirmed that there are no confounding factors in the relationship between AIDs and PCa. Genetic variation, specifically horizontal pleiotropy, can introduce bias in the causal estimates instead of separate exposures. This circumstance contradicts the assumptions of Mendelian randomization (MR). In order to address this issue, we utilised three separate analytical approaches in the MR analysis, each relying on a unique horizontal pleiotropy framework. The credibility of the findings is enhanced by comparing the results obtained from these three methods, as the consistency across approaches improves. The main analysis used the inverse variance-weighting (IVW) method^27^, which provided the most accurate estimates but made the assumption that all SNPs were valid IVs. If one SNP does not adhere to the IVs assumption, the random-defect IVW will be employed to produce a bias by considering potential heterogeneity and assigning weights to each rate based on their standard error. To meet the requirements of a valid instrumental variable, the weighted median approach requires a minimum of 50% SNPs^28^. After sorting the weights, we calculated the median of the connected distribution function using the results from our experiments with the included SNPs. Furthermore, in the absence of pleiotropic effects, MR-Egger regression can be utilised to obtain an estimate of the effect if the genetic instrument is independent^29^. The pleiotropic effect was assessed using the intercept of MR-Egger. Moreover, in the absence of a significant deviation from zero in MR-Egger's intercept, it is not possible to demonstrate a directional multiplicative effect^30^.

### Sensitivity analysis

**Figure 2:**
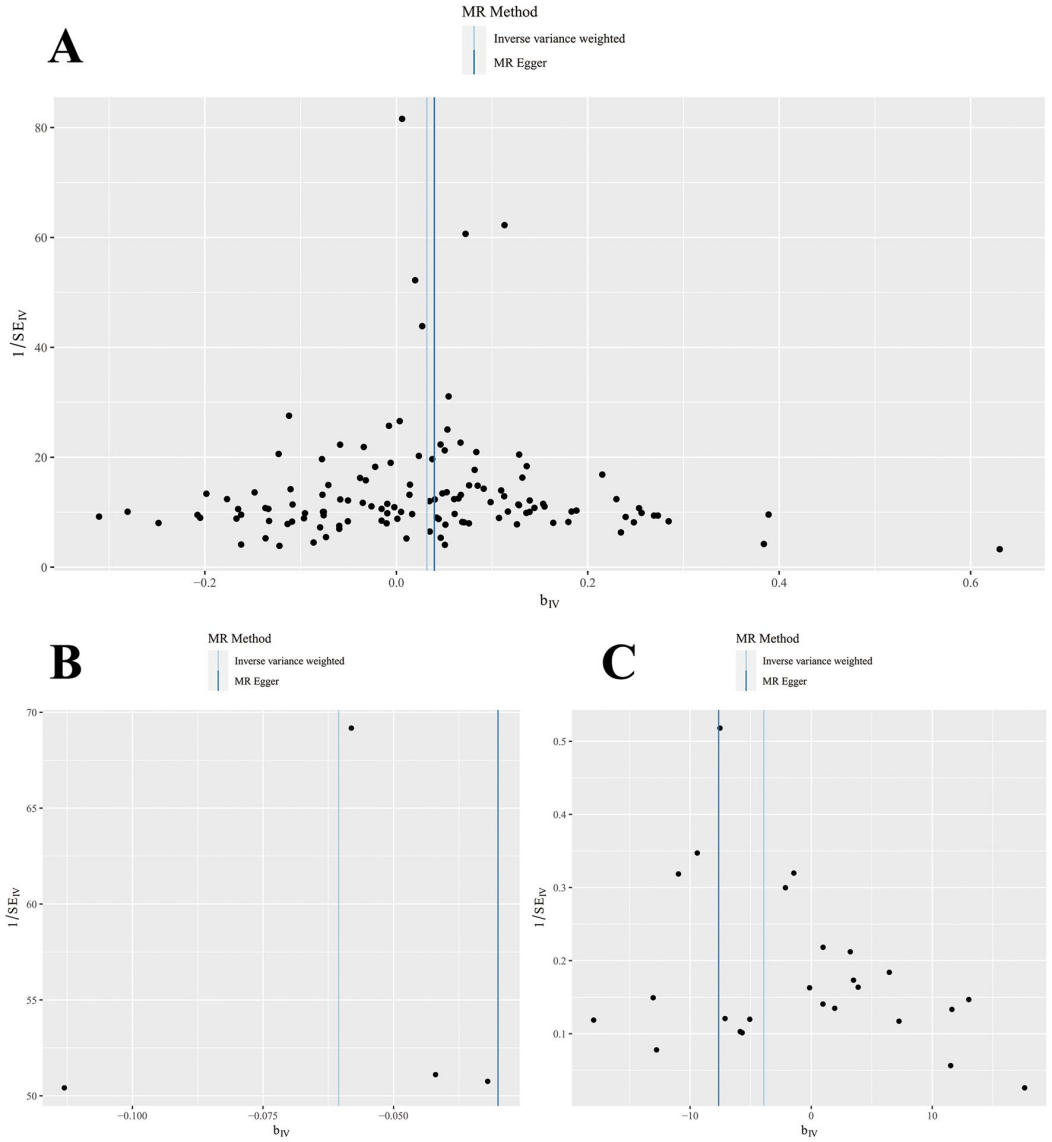
The funnel plot of the causal effect of AIDs SNPs on PCa. (The MR-Egger and IVW were used for the analyses. Individual SNP was delineated in the background. **A** RA; **B** SLE; **C** Hyperthyroidism).

**Figure 3:**
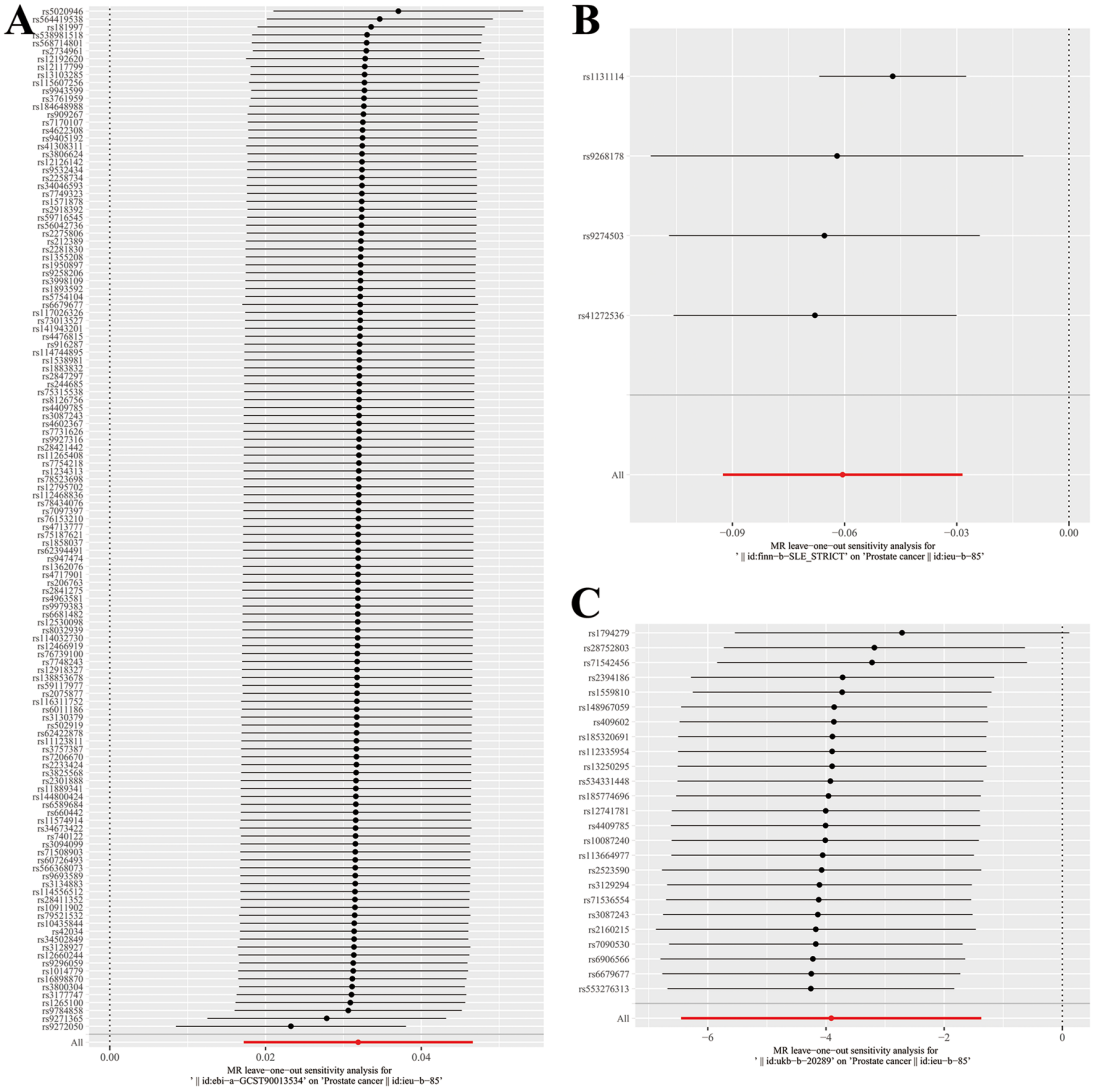
The leave-one-out plot of the causal effect of AIDs SNPs on PCa. (**A** RA; **B** SLE; **C** Hyperthyroidism).

By showing a solitary Wald ratio for each single nucleotide polymorphism (SNP), funnel plots can effectively demonstrate the directional level pleiotropy of instrumental variables (IVs). Nevertheless, evaluating horizontal pleiotropy using funnel plots becomes difficult as a result of the restricted number of IVs considered in the analysis. Notably, the funnel plot illustrated a nearly balanced pattern (Fig. 2). To explore the potential prejudice or impact of specific SNPs on the IVW analysis estimates, leave-one-out analyses were performed. These analyses involved running meta-analyses using the reanalyzed IVW results after excluding one SNP at a time. After removing each SNP, a subsequent MR analysis was conducted systematically for the remaining SNPs. Surprisingly, the findings consistently demonstrated a notable cause-and-effect connection among all the SNPs (Fig. 3). In the realm of Mendelian randomization (MR) analysis, the second proposition suggests that single nucleotide polymorphisms (SNPs) influence the exposure of interest exclusively by modifying it, without any interference from other confounding pathways. MR-Egger regression was used to obtain the intercept and its corresponding p-value for evaluating directional multidirectionality. Importantly, the MR-Egger regression intercept did not show any indication of horizontal pleiotropy (*p* > 0.05), which further strengthens the argument that pleiotropy did not introduce any distortion to the estimation of the causal effect.

Furthermore, a thorough examination of the published genome-wide association study (GWAS) data uncovered no notable connections between the SNPs linked to AIDs and any other characteristics, with the exception of AIDs itself. This finding signifies that the assumptions underlying the third MR analysis were upheld without violation. Hence, there is no indication indicating substantial connections between the hereditary tools of the linked SNPs and other characteristics on a comprehensive genomic level. This backs our third MR hypothesis, which is improbable to be breached in our investigation^26^. The Benjamini–Hochberg method that controls the false discovery rate (FDR) was applied to correct for multiple testing. After correction, we found that SLE, RA, and hyperthyroidism all exhibit significant causal relationships with PCa (FDR < 0.05). The "Two sample MR" (version 0.5. 6) software package was applied for MR and sensitivity analysis in R (version 4.2. 1)^31^.

### Human and animal rights

This study is not including human or animal subjects.

## Results

### Instrumental variables for autoimmune diseases

**Figure 4:**
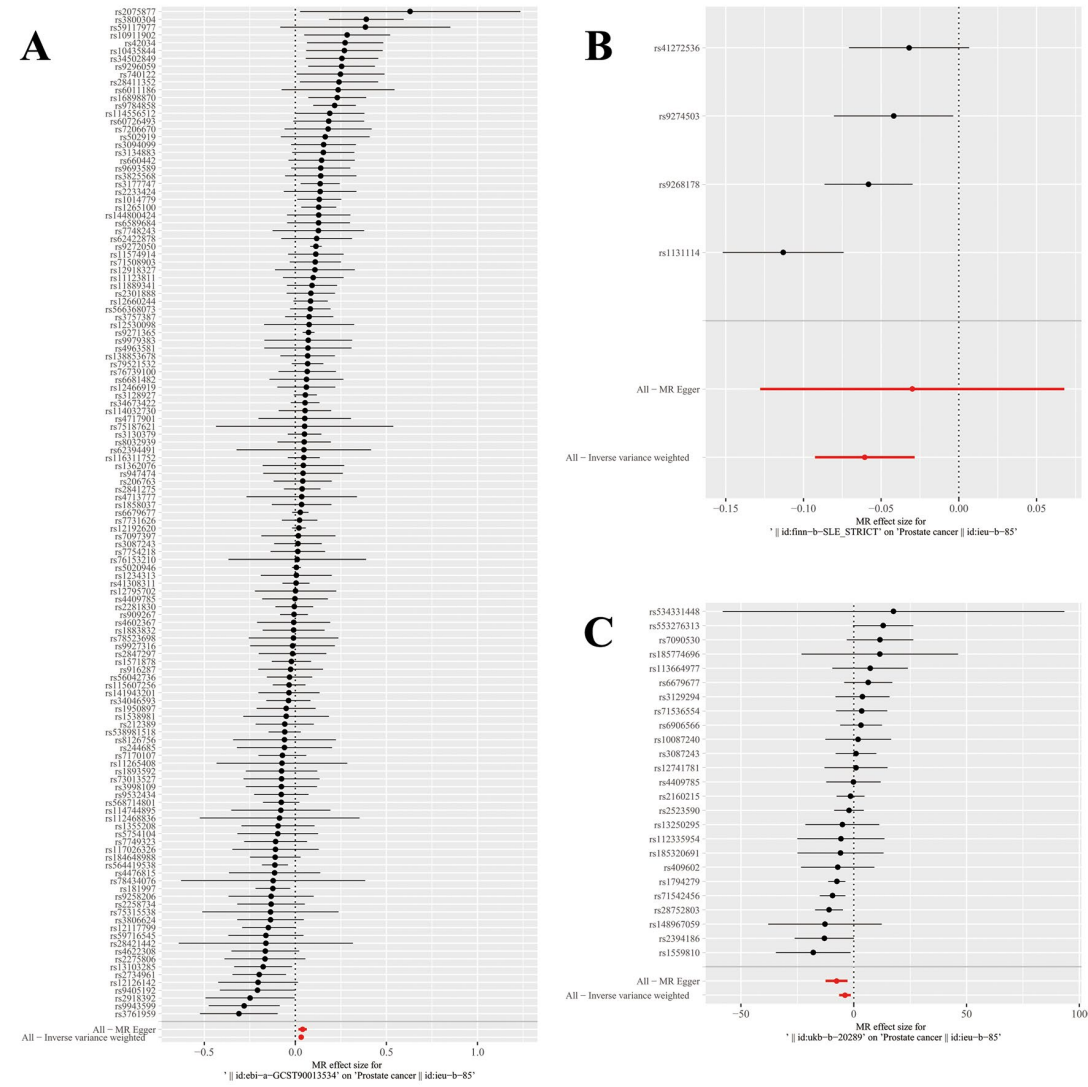
The forest plot of the causal effect of AIDs SNPs on PCa. (**A** RA; **B** SLE; **C** Hyperthyroidism).

**Figure 5:**
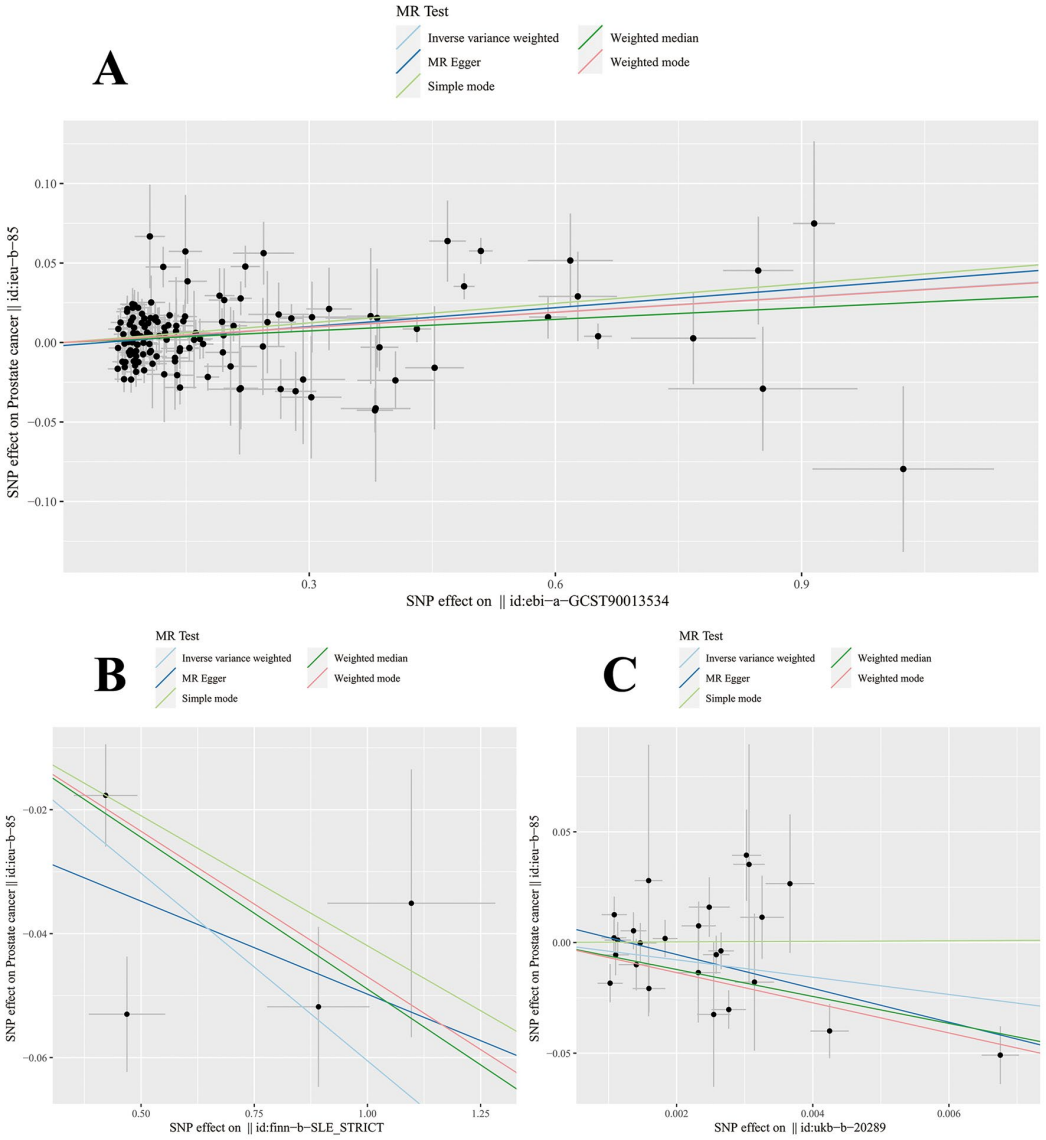
The scatter plot of the causal effect of AIDs SNPs on PCa. The weighted median, MR-Egger, IVW, weighted mode methods and simple mode were used for the analyses. The slope of each line represents the causal estimates for each method. (**A** RA; **B** SLE; **C** Hyperthyroidism) SNP, single nucleotide polymorphism; MR, Mendelian.

The genetic variations associated with AIDs are displayed in (Supplementary Table 1). All genetic tools linked to AIDs were significant at a genome-wide level (*p* < 5 × 10E−8, F > 10). The impact of each genetic variant on PCa is demonstrated through forest plots and scatter plots (Figs. 4, 5).

### Mendelian randomization analyses for prostate *cancer*

We assessed the association between AIDs and PCa using MR-Egger, weighted median, IVW, and weighted mode regression (Table 1). The results of our study indicate a slight elevation of developing PCa in patients with RA (OR = 1.03; 95% CI 1.01–1.04, *p* = 2*10E−5). Additionally, there is evidence suggesting a lower risk of PCa in individuals diagnosed with SLE. (OR = 0.94; 95% CI 0.91–0.97, *p* = 2*10E−4). The results indicate a reduced likelihood of PCa in individuals with hyperthyroidism (OR = 0.02; 95% CI 0.001–0.2, *p* = 2*10E−3).

**Table 1:** MR analyses of the causal relationship between three common AIDs PCa.

Exposure	Outcome	Number of SNPs	Methods	BETA	SE	Pval	OR (95% CI)
RA	PCa	129	MR Egger	0.0397	0.0117	8.90E−04	1.0405 (1.0170–1.0646)
			Weighted median	0.0243	0.0097	1.28E−02	1.0246 (1.0043–1.0452)
			IVW	0.0319	0.0075	2.13E−05	1.0324 (1.0173–1.0477)
			Weighted mode	0.0316	0.0101	2.12E−03	1.0321 (1.0113–1.0533)
SLE	PCa	4	MR Egger	− 0.03	0.0499	6.09E−01	0.9704 (0.8800–1.0702)
			Weighted median	− 0.0489	0.0126	1.00E−04	0.9522 (0.9292–0.9758)
			IVW	− 0.0605	0.0163	2.14E−04	0.9413 (0.9116–0.9719)
			Weighted mode	− 0.047	0.0147	4.98E−02	0.9541 (0.9286–0.9804)
Hyperthyroidism	PCa	25	MR Egger	− 7.6418	2.4763	5.22E−03	0.0005 (0.0000–0.0615)
			Weighted median	− 6.0928	1.5426	7.83E−05	0.0023 (0.0001–0.0497)
			IVW	− 3.9118	1.2964	2.55E−03	0.0200 (0.0016–0.2539)
			Weighted mode	− 6.8099	1.5768	2.35E−04	0.0011 (0.0000–0.0258)

### Assessment of MR assumptions

In our study, the selection of SNPs was based on the genome-wide significance threshold of *p* < 5 × 10E−8) in these analyses provided evidence for the absence of directional pleiotropy of PCa, suggesting that the second MR assumption was not violated. Additionally, the MR heterogeneity test showed no heterogeneity in our positive outcomes. In summary, the rigorous assessment of the three fundamental assumptions in MR analysis was conducted in our study. The results suggested that the selected SNPs were appropriate as genetic instruments, and the relationships between genetically predicted AIDs and PCa were not confounded by potential confounders or mediators.

## Discussion

The objective of this study was to confirm the causal connection between three prevalent autoimmune diseases, namely systemic lupus erythematosus (SLE), overactive thyroid (hyperthyroidism), rheumatoid arthritis (RA), and prostate cancer (PCa). Earlier research has already established a cause-and-effect connection between systemic lupus erythematosus (SLE) and hyperthyroidism, along with prostate cancer (PCa)^20,21^. It's important to point out that even though our study and the research by Xu and Chen both focus on people from European backgrounds, we found the odds ratio (OR_IVW) to be 0.020, in contrast to Xu and Chen's finding of 0.859. This discrepancy could be due to a combination of factors, including the varying sizes and geographical origins of the sample populations we studied. These regional differences could introduce variability in genetic and environmental factors affecting our findings. Moreover, the samples collected from these populations were analyzed by different biotech companies, potentially leading to variations in the data due to differing methodologies or technological capabilities. Alongside these considerations, variations in the selection of instrumental variables and the methodological approaches we each used, as well as the specific datasets analyzed, further contribute to the observed differences. This shows we need more research, like meta-analyses, to better understand these differences and what they mean for how genetics might play a role in these diseases. This study aims to investigate the potential causal link between RA and PCa through MR analysis. To our understanding, this is the initial Mendelian randomization study that comprehensively evaluates the impact of genetic vulnerability on the likelihood of developing RA with PCa, involving two distinct samples.

Numerous prior investigations have demonstrated a correlation between RA and a higher susceptibility to lung cancer^32^, lymphoma malignancy^33^, ovarian malignancy^34^, Cervical Cancer^35^ and other malignancies, in comparison to the overall population. are at increased risk. With regard to PCa, there are not many previous studies. Based on 17 studies, a meta-analysis found that RA patients have a 1.15 SIR (with a 95% confidence interval of 0.98–1.34) for PCa disorders compared to the general population^33^. However, the SIR ranges in the individual studies included in this meta-analysis varied greatly, indicating substantial heterogeneity and unreliability in the study results. In a recent 8-year cohort study involving a significant number of US veteran men, which is also the most extensive research on PCa disorders in RA so far, it was found that the risk of PCa disorders was only slightly higher in patients with RA. Nevertheless, the research failed to present proof endorsing a cause-and-effect connection between RA and PCa ailments^22^. The majority of the evidence associating RA with PCa susceptibility stems from observational investigations, which could be hindered by confounding variables, study length, lifestyle, environmental influences, and reverse causality^36^. Hence, it is inadequate to reach a sound conclusion.

The utilisation of genetic variation as instrumental variables (IVs) in Mendelian randomization aids in addressing these difficulties by eliminating confounders and inferring correlated characteristics between cause and effect^37,38^. Mendel's second principle, asserting that alleles are haphazardly distributed during reproduction, can be seen as nature's randomised experiment, where genetic diversity serves as a natural randomizing element^39^. Genetic variation is determined at conception and is relatively stable and largely independent of environmental factors. Furthermore, various statistical techniques have been utilised in MR studies to investigate causal impacts^40^. In our MR study, we discovered potential signs of a slightly increased likelihood of prostate cancer in patients with rheumatoid arthritis (RA), which could offer valuable knowledge for future extensive and enduring cohort studies of excellent quality exploring the possible connection between RA and PCa.

Nevertheless, the Main potential mechanisms remain uncertain and could be influenced by multiple factors. RA, also known as rheumatoid arthritis, is a persistent autoimmune disorder that causes widespread inflammation throughout the body^14^. Previous studies have reported the chronic inflammatory pathogenesis of many cancers, including PCa^41–43^. Based on studies, it is inferred that individuals with rheumatoid arthritis (RA) may have an increased susceptibility to prostate cancer (PCa) due to the common characteristic of persistent inflammation. C-reactive protein (CRP) could potentially serve as a significant mediator in this mechanism. The cytokine IL-6 has been found to play a vital part in the development of RA^44^, activating pathways that promote inflammation throughout the body. As an important regulator, IL-6 promotes the production of the acute phase reactant CRP in response to inflammation. Thus, CRP may be regarded as a consequence of IL-6 and functions as a crucial indicator of widespread inflammation in RA^45,46^. Additional investigation is required to comprehensively comprehend the precise mechanism by which CRP contributes to the incidence and progression of PCa, despite the existing correlation between elevated levels of circulating CRP and heightened PCa risk^47^.

Second, anti-inflammatory drugs (AIMs) are medications that reduce inflammation and its symptoms. In order to suppress inflammatory responses through various mechanisms, these medications can be classified into various categories, including non-steroidal anti-inflammatory drugs (NSAIDs), glucocorticoids, and immunosuppressants, such as disease-modifying anti-rheumatic drugs (DMARDs) and biologic disease-modifying anti-rheumatic drugs (bDMARDs). "In a study that compared individuals with and without prostate cancer in a population, the utilisation of AIMs was linked to a higher likelihood of developing PCa (odds ratio 1.26; 95% confidence interval 1.24–1.29), especially when it came to non-aspirin NSAIDs^48^. The precise cause for this elevated risk remains uncertain and could be influenced by the specific dosage and duration of drug usage. Regular use of glucocorticoids is connected to an increased risk of PCa^48^, potentially due to their immunosuppressive effects that weaken defence mechanisms and lead to metabolic changes. The continuous use of certain medications can lead to increased blood sugar levels (hyperglycemia) and stimulate the growth of prostate cells through the androgen receptor^49–51^. Studies on RA patients receiving DMARDS or bDMARDS indicate that anti-inflammatory therapy is not likely to be associated with an increased risk of cancer^52–54^. Overall, the immunosuppressive treatment for RA is not expected to significantly raise the risk of prostate cancer, especially with the commonly used RA treatments.

Furthermore, the potential causes for the heightened susceptibility to cancer in individuals with RA might involve common genetic factors. The underlying biological mechanism may be caused by a flaw in this organism's regular immune surveillance system, which is unable to successfully deal with the abnormal cells created during cell reproduction. A healthy immune system can eliminate these abnormal cells before they develop into clinical cancer. Nonetheless, in people with acquired immunodeficiency syndrome (AIDs) like RA, this monitoring procedure might be compromised, heightening the likelihood of cancer development^55^. The biochemical mechanisms that connect RA and PCa are more intricate than inflammation and will necessitate further investigation for confirmation.

In line with prior Mendelian randomization investigations, our study also validated a cause-and-effect association between hyperthyroidism and SLE with PCa, while showcasing a safeguarding impact. Moreover, our study employed an independent database for the outcome factors, adding additional strengths to our analysis. By validating and adding to this separate data source, we acquired broader and dependable findings that further reinforced the proof of a connection between overactive thyroid, systemic lupus erythematosus, and prostate cancer. Unlike previous observational studies, our MR analysis demonstrated that hyperthyroidism has a beneficial impact on the risk of developing PCa. It is worth mentioning that a significant Israeli study involving 375,635 individuals without any previous cancer history and a total of 23,808 diagnosed tumors during a median follow-up of 10 years found a correlation between TSH levels in hyperthyroidism and an elevated likelihood of PCa. in the meantime, hypothyroid TSH levels were related with a lower incidence of PCa^56^. Likewise, Mondul et al.^57^ discovered a lower incidence of PCa in males with hypothyroidism. The community dwelling population surveyed by Chan et al. The study indicated that higher levels of the thyroid hormone FT4 were linked to an elevated chance of PCa^58–60^. This is due to the interaction between thyroid hormones and the plasma membrane integrin αVβ3, which leads to the proliferation of tumor cells and angiogenesis^61^. On the other hand, there are theories suggesting that thyroid hormones may actually have a protective effect against cancer^62^. The age of the patient might play a role in this, as hyperthyroidism is associated with an increased risk of cancer in younger patients but may be protective in older patients^56^. Unfortunately, there is currently a lack of observational studies supporting a reduced risk of PCa, and the underlying biological mechanisms require further investigation for validation.

Numerous studies have verified that the androgen reliance of PCa cells poses a significant hazard for the emergence of this cancer^63^. Conversely, SLE patients exhibit notably diminished levels of androgens (testosterone and androstenedione) in comparison to healthy individuals^64^. Consequently, the primary mechanism at play might be the reduction in PCa risk due to the low testosterone levels observed in SLE patients. Interestingly, the utilisation of immunosuppressive drugs in individuals with systemic lupus erythematosus (SLE) was discovered to be linked with a heightened susceptibility to prostate cancer (odds ratio [OR] = 1.1073; 95% confidence interval [CI] = 1.0538–1.1634; *P* < 0.001)^20,21^, Conversely, the administration of immunosuppressive drugs in rheumatoid arthritis (RA) holds minimal importance, necesitating further investigation into the underlying causes.

This MR study is assessing three autoimmune diseases: hyperthyroidism, SLE, and RA. Additionally, it is the inaugural MR study examining the relationship between RA and PCa. This finding has important implications for clinical practise, providing guidance for patient management and prevention strategies, and helping physicians better focus on their patients' PCa risk and take early screening and treatment measures. Furthermore, these discoveries will broaden the perspectives of prostate cancer prevention studies and offer fresh insights for further investigation into biomarkers and therapeutic targets for prostate cancer. Increasing public knowledge about the correlation between AIDs and PCa can boost patients' health consciousness and encourage their active involvement in cancer prevention and early detection. Ultimately, our discoveries will establish a basis for making decisions regarding public health and creating more specific health strategies to reduce the occurrence and death rate of PCa, while simultaneously enhancing the prognosis and quality of life for patients.

Our study's primary advantage is its two-sample MR study design, which reduces the impact of unobserved confounding and reverse causation, thus improving causal inference. Furthermore, in contrast to observational research, our approach minimizes the possibility of unreliable outcomes by incorporating genotype as an intermediary factor, thereby enhancing control over confounding variables. Furthermore, our focus on data from European populations ensures potential immunity to demographic stratification, improving the study's internal consistency and comparability. Nevertheless, it is crucial to recognise certain constraints in our research. The estimation accuracy may have been affected and the likelihood of incorrect negative results may have increased due to the relatively low number of cancer cases in the MR analyses compared to the control group. Additionally, our in-depth understanding of individual differences was limited as we were unable to perform patient-level analyses stratified by factors like age, gender, and ethnicity, due to the absence of raw data. In conclusion, the constraints of the accessible data prevented us from obtaining conclusive proof of particular tumourigenesis mechanisms, instead, we only observed associations.

In summary, this research offers significant fundamental proof for investigating the cause-and-effect connection among hyperthyroidism, SLE, RA, and PCa. Gaining insights into the intricate connections between AIDs and cancer risk is crucial, as it can ultimately enhance clinical management and aid in the development of public health strategies.

### Supplementary Information

Supplementary Information.

## Data Availability

All data relevant to the study are included in the article.The exposure datasets generated in this research are available in the MR-base repository, [https://gwas.mrcieu.ac.uk/datasets/]. Specifically, “ebi-a-GCST90013534” for RA, “finn-b-SLE_STRICT” for SLE, “ukb-b-20289” for hyperthyroidism. The outcome datasets named used in this research were acquired from the MR-base repository, [https://gwas.mrcieu.ac.uk/datasets/], “ieu-b-85”for RA.
