# Supplementary material for: The association between three prevalent autoimmune disorders and the likelihood of developing prostate cancer: a Mendelian randomization study
Source: Sci Rep. 2024 May 23;14:11755. doi: 10.1038/s41598-024-62716-6 (PMC11116512; doi:10.1038/s41598-024-62716-6)
Supplement: Supplementary file 1 — Supplementary Information. [file 41598_2024_62716_MOESM1_ESM.docx]

| Exposure | SNP | chr | EA | OA | SNP-EXPOSURE Association | | | | SNP-OUTCOME Association | | | |
| --- | --- | --- | --- | --- | --- | --- | --- | --- | --- | --- | --- | --- |
|  |  |  |  |  | beta | se | eaf | pval | beta | se | eaf | pval |
| RA | rs1014779 | 6 | A | G | 0.1298 | 0.0118 | 0.2821 | 2.78E-28 | 0.0171 | 0.2821 | 0.0080 | 3.21E-02 |
|  | rs10435844 | 9 | T | G | -0.0784 | 0.0121 | 0.4942 | 9.73E-11 | -0.0211 | 0.4942 | 0.0084 | 1.18E-02 |
|  | rs10911902 | 1 | T | C | -0.0847 | 0.0152 | 0.1246 | 2.36E-08 | -0.0241 | 0.1246 | 0.0102 | 1.79E-02 |
|  | rs11123811 | 2 | C | T | -0.0995 | 0.0114 | 0.6512 | 2.01E-18 | -0.0098 | 0.6512 | 0.0084 | 2.44E-01 |
|  | rs112468836 | 6 | A | G | -0.1364 | 0.0235 | 0.1287 | 6.18E-09 | 0.0118 | 0.1287 | 0.0305 | 6.99E-01 |
|  | rs11265408 | 1 | T | G | 0.2042 | 0.0320 | 0.0642 | 1.69E-10 | -0.0150 | 0.0642 | 0.0373 | 6.88E-01 |
|  | rs114032730 | 6 | A | G | 0.3034 | 0.0423 | 0.0168 | 7.82E-13 | 0.0160 | 0.0168 | 0.0223 | 4.72E-01 |
|  | rs114556512 | 16 | A | G | -0.0962 | 0.0174 | 0.3758 | 3.41E-08 | -0.0181 | 0.3758 | 0.0094 | 5.31E-02 |
|  | rs114744895 | 6 | C | A | -0.2926 | 0.0512 | 0.1654 | 1.11E-08 | 0.0233 | 0.1654 | 0.0405 | 5.65E-01 |
|  | rs115607256 | 6 | A | G | -0.8528 | 0.1152 | 0.0097 | 1.32E-13 | 0.0291 | 0.0097 | 0.0390 | 4.56E-01 |
|  | rs11574914 | 9 | A | G | 0.1153 | 0.0149 | 0.1513 | 9.92E-15 | 0.0130 | 0.1513 | 0.0089 | 1.46E-01 |
|  | rs116311752 | 6 | A | G | 0.6273 | 0.0477 | 0.0122 | 1.86E-39 | 0.0290 | 0.0122 | 0.0281 | 3.02E-01 |
|  | rs11677109 | 2 | A | T | -0.0877 | 0.0157 | 0.4837 | 2.40E-08 | -0.0025 | 0.4837 | 0.0094 | 7.91E-01 |
|  | rs117026326 | 7 | T | C | 0.3810 | 0.0424 | 0.1113 | 2.45E-19 | -0.0415 | 0.1113 | 0.0460 | 3.67E-01 |
|  | rs11889341 | 2 | T | C | 0.1466 | 0.0129 | 0.2112 | 4.32E-30 | 0.0134 | 0.2112 | 0.0103 | 1.92E-01 |
|  | rs12117799 | 1 | T | C | -0.1391 | 0.0207 | 0.0922 | 1.99E-11 | 0.0206 | 0.0922 | 0.0102 | 4.37E-02 |
|  | rs12126142 | 1 | A | G | -0.0751 | 0.0116 | 0.2969 | 1.01E-10 | 0.0154 | 0.2969 | 0.0084 | 6.58E-02 |
|  | rs12192620 | 6 | A | G | 0.4313 | 0.0173 | 0.5425 | 8.99E-138 | 0.0085 | 0.5425 | 0.0083 | 3.04E-01 |
|  | rs1234313 | 1 | G | A | 0.0797 | 0.0133 | 0.6075 | 1.90E-09 | 0.0004 | 0.6075 | 0.0079 | 9.60E-01 |
|  | rs12466919 | 2 | T | C | 0.1025 | 0.0152 | 0.1733 | 1.59E-11 | 0.0062 | 0.1733 | 0.0083 | 4.55E-01 |
|  | rs12530098 | 6 | T | C | 0.1382 | 0.0204 | 0.1223 | 1.35E-11 | 0.0105 | 0.1223 | 0.0174 | 5.46E-01 |
|  | rs1265100 | 6 | G | A | 0.2166 | 0.0134 | 0.4327 | 5.66E-59 | 0.0278 | 0.4327 | 0.0106 | 8.56E-03 |
|  | rs12660244 | 6 | A | G | -0.6182 | 0.0518 | 0.1313 | 8.22E-33 | -0.0516 | 0.1313 | 0.0295 | 8.05E-02 |
|  | rs12795702 | 11 | G | A | -0.0845 | 0.0144 | 0.5163 | 4.09E-09 | -0.0001 | 0.5163 | 0.0096 | 9.92E-01 |
|  | rs12918327 | 16 | T | C | 0.0867 | 0.0157 | 0.1118 | 3.04E-08 | 0.0093 | 0.1118 | 0.0097 | 3.36E-01 |
|  | rs13103285 | 4 | T | C | 0.0989 | 0.0131 | 0.4018 | 4.29E-14 | -0.0175 | 0.4018 | 0.0080 | 2.86E-02 |
|  | rs1355208 | 2 | G | A | 0.0818 | 0.0119 | 0.6377 | 6.77E-12 | -0.0078 | 0.6377 | 0.0083 | 3.50E-01 |
|  | rs1362076 | 6 | T | G | -0.3749 | 0.0291 | 0.0920 | 5.74E-38 | -0.0166 | 0.0920 | 0.0428 | 6.98E-01 |
|  | rs138853678 | 6 | C | G | -0.2628 | 0.0368 | 0.3965 | 9.81E-13 | -0.0177 | 0.3965 | 0.0200 | 3.76E-01 |
|  | rs141943201 | 6 | G | A | -0.4527 | 0.0357 | 0.1250 | 7.15E-37 | 0.0159 | 0.1250 | 0.0387 | 6.81E-01 |
|  | rs144800424 | 1 | T | C | 0.0959 | 0.0144 | 0.3663 | 2.52E-11 | 0.0123 | 0.3663 | 0.0085 | 1.48E-01 |
|  | rs1538981 | 10 | T | C | 0.0671 | 0.0114 | 0.3568 | 4.42E-09 | -0.0034 | 0.3568 | 0.0080 | 6.73E-01 |
|  | rs1571878 | 6 | T | C | -0.1539 | 0.0116 | 0.3952 | 4.13E-40 | 0.0034 | 0.3952 | 0.0084 | 6.87E-01 |
|  | rs16898870 | 6 | C | T | -0.2444 | 0.0371 | 0.4860 | 4.72E-11 | -0.0562 | 0.4860 | 0.0197 | 4.40E-03 |
|  | rs181997 | 6 | A | G | -0.1764 | 0.0139 | 0.1607 | 1.08E-36 | 0.0217 | 0.1607 | 0.0086 | 1.12E-02 |
|  | rs184648988 | 6 | C | G | -0.2653 | 0.0220 | 0.1992 | 1.75E-33 | 0.0293 | 0.1992 | 0.0187 | 1.18E-01 |
|  | rs1858037 | 2 | A | T | -0.1012 | 0.0131 | 0.4074 | 1.14E-14 | -0.0035 | 0.4074 | 0.0084 | 6.78E-01 |
|  | rs1883832 | 20 | C | T | 0.1052 | 0.0127 | 0.7483 | 1.13E-16 | -0.0010 | 0.7483 | 0.0091 | 9.13E-01 |
|  | rs1893592 | 21 | C | A | -0.0976 | 0.0132 | 0.4611 | 1.48E-13 | 0.0074 | 0.4611 | 0.0097 | 4.47E-01 |
|  | rs1950897 | 14 | T | C | 0.1069 | 0.0144 | 0.5178 | 1.02E-13 | -0.0054 | 0.5178 | 0.0088 | 5.40E-01 |
|  | rs206763 | 6 | A | G | -0.3828 | 0.0629 | 0.0094 | 1.19E-09 | -0.0154 | 0.0094 | 0.0311 | 6.20E-01 |
|  | rs2075877 | 21 | A | G | 0.1058 | 0.0183 | 0.3597 | 7.99E-09 | 0.0667 | 0.3597 | 0.0326 | 4.09E-02 |
|  | rs212389 | 6 | A | G | 0.1058 | 0.0147 | 0.4297 | 6.66E-13 | -0.0062 | 0.4297 | 0.0086 | 4.71E-01 |
|  | rs2233424 | 6 | T | C | 0.1964 | 0.0187 | 0.1933 | 6.49E-26 | 0.0267 | 0.1933 | 0.0199 | 1.80E-01 |
|  | rs2258734 | 1 | A | G | -0.0921 | 0.0123 | 0.2989 | 6.04E-14 | 0.0123 | 0.2989 | 0.0087 | 1.57E-01 |
|  | rs2275806 | 10 | A | G | -0.0725 | 0.0122 | 0.4361 | 2.51E-09 | 0.0121 | 0.4361 | 0.0082 | 1.40E-01 |
|  | rs2281830 | 6 | A | G | 0.1708 | 0.0125 | 0.3942 | 2.06E-42 | -0.0010 | 0.3942 | 0.0090 | 9.12E-01 |
|  | rs2301888 | 1 | A | G | -0.1282 | 0.0121 | 0.3847 | 3.75E-26 | -0.0109 | 0.3847 | 0.0087 | 2.08E-01 |
|  | rs244685 | 5 | G | T | -0.0890 | 0.0144 | 0.7884 | 6.04E-10 | 0.0053 | 0.7884 | 0.0118 | 6.55E-01 |
|  | rs2734961 | 6 | T | C | 0.1426 | 0.0198 | 0.3724 | 6.14E-13 | -0.0283 | 0.3724 | 0.0107 | 8.01E-03 |
|  | rs28411352 | 1 | T | C | 0.0914 | 0.0136 | 0.1803 | 1.66E-11 | 0.0219 | 0.1803 | 0.0100 | 2.85E-02 |
|  | rs2841275 | 14 | C | A | 0.1617 | 0.0179 | 0.6245 | 1.71E-19 | 0.0061 | 0.6245 | 0.0082 | 4.58E-01 |
|  | rs28421442 | 2 | A | T | -0.1234 | 0.0214 | 0.1569 | 7.86E-09 | 0.0200 | 0.1569 | 0.0301 | 5.06E-01 |
|  | rs2847297 | 18 | G | A | 0.0903 | 0.0119 | 0.5197 | 2.65E-14 | -0.0014 | 0.5197 | 0.0085 | 8.70E-01 |
|  | rs2918392 | 5 | C | T | 0.0668 | 0.0122 | 0.6939 | 4.62E-08 | -0.0166 | 0.6939 | 0.0083 | 4.64E-02 |
|  | rs3087243 | 2 | A | G | -0.1261 | 0.0124 | 0.2260 | 3.32E-24 | -0.0018 | 0.2260 | 0.0084 | 8.30E-01 |
|  | rs3094099 | 6 | C | T | -0.1907 | 0.0181 | 0.2971 | 6.45E-26 | -0.0295 | 0.2971 | 0.0173 | 8.80E-02 |
|  | rs3128927 | 6 | T | C | -0.2783 | 0.0144 | 0.1453 | 1.20E-82 | -0.0152 | 0.1453 | 0.0090 | 8.97E-02 |
|  | rs3130379 | 6 | C | T | 0.2078 | 0.0165 | 0.8990 | 2.64E-36 | 0.0105 | 0.8990 | 0.0098 | 2.83E-01 |
|  | rs3134883 | 10 | A | G | 0.0991 | 0.0125 | 0.2065 | 1.98E-15 | 0.0152 | 0.2065 | 0.0086 | 7.72E-02 |
|  | rs3177747 | 6 | A | G | 0.4685 | 0.0223 | 0.0993 | 5.94E-98 | 0.0638 | 0.0993 | 0.0255 | 1.24E-02 |
|  | rs34046593 | 4 | A | G | 0.1422 | 0.0170 | 0.1573 | 7.17E-17 | -0.0054 | 0.1573 | 0.0088 | 5.38E-01 |
|  | rs34502849 | 11 | A | G | -0.0851 | 0.0140 | 0.2473 | 1.07E-09 | -0.0218 | 0.2473 | 0.0086 | 1.16E-02 |
|  | rs34536443 | 19 | C | G | -0.3801 | 0.0474 | 0.5217 | 1.08E-15 | -0.0398 | 0.5217 | 0.0222 | 7.27E-02 |
|  | rs34673422 | 6 | C | A | 0.8472 | 0.0428 | 0.2932 | 2.69E-87 | 0.0453 | 0.2932 | 0.0339 | 1.81E-01 |
|  | rs3757387 | 7 | C | T | 0.1236 | 0.0137 | 0.5585 | 1.87E-19 | 0.0094 | 0.5585 | 0.0083 | 2.58E-01 |
|  | rs3761959 | 1 | T | C | 0.0744 | 0.0115 | 0.3069 | 9.64E-11 | -0.0231 | 0.3069 | 0.0081 | 4.23E-03 |
|  | rs3800304 | 6 | A | G | 0.1221 | 0.0214 | 0.0581 | 1.21E-08 | 0.0475 | 0.0581 | 0.0128 | 1.97E-04 |
|  | rs3806624 | 3 | G | A | 0.0863 | 0.0131 | 0.7654 | 3.94E-11 | -0.0118 | 0.7654 | 0.0080 | 1.42E-01 |
|  | rs3825568 | 14 | T | C | 0.0812 | 0.0118 | 0.4254 | 6.10E-12 | 0.0113 | 0.4254 | 0.0081 | 1.63E-01 |
|  | rs3998109 | 6 | A | G | -0.0848 | 0.0146 | 0.1708 | 6.68E-09 | 0.0065 | 0.1708 | 0.0084 | 4.41E-01 |
|  | rs41308311 | 6 | A | G | -0.7681 | 0.0760 | 0.0104 | 5.15E-24 | -0.0027 | 0.0104 | 0.0289 | 9.26E-01 |
|  | rs42034 | 7 | G | A | 0.0871 | 0.0153 | 0.4650 | 1.28E-08 | 0.0238 | 0.4650 | 0.0093 | 1.05E-02 |
|  | rs4409785 | 11 | C | T | 0.0982 | 0.0170 | 0.4338 | 7.85E-09 | -0.0002 | 0.4338 | 0.0090 | 9.82E-01 |
|  | rs4476815 | 6 | C | G | -0.3025 | 0.0368 | 0.1284 | 1.92E-16 | 0.0344 | 0.1284 | 0.0386 | 3.73E-01 |
|  | rs4602367 | 3 | G | A | 0.0750 | 0.0117 | 0.6972 | 1.76E-10 | -0.0007 | 0.6972 | 0.0077 | 9.27E-01 |
|  | rs4622308 | 12 | T | C | 0.0878 | 0.0125 | 0.4615 | 2.21E-12 | -0.0145 | 0.4615 | 0.0083 | 8.10E-02 |
|  | rs4713777 | 6 | A | G | 0.1172 | 0.0161 | 0.5022 | 3.46E-13 | 0.0041 | 0.5022 | 0.0181 | 8.21E-01 |
|  | rs4717901 | 7 | C | A | 0.2490 | 0.0349 | 0.1939 | 9.52E-13 | 0.0128 | 0.1939 | 0.0322 | 6.91E-01 |
|  | rs4963581 | 12 | A | G | 0.0856 | 0.0156 | 0.5109 | 3.75E-08 | 0.0059 | 0.5109 | 0.0104 | 5.72E-01 |
|  | rs5020946 | 6 | T | G | 0.6519 | 0.0169 | 0.4122 | 1.00E-200 | 0.0039 | 0.4122 | 0.0080 | 6.26E-01 |
|  | rs502919 | 10 | C | T | 0.0829 | 0.0134 | 0.4349 | 6.17E-10 | 0.0136 | 0.4349 | 0.0103 | 1.86E-01 |
|  | rs538981518 | 6 | T | C | 0.4052 | 0.0465 | 0.0798 | 3.03E-18 | -0.0238 | 0.0798 | 0.0182 | 1.91E-01 |
|  | rs56042736 | 6 | G | A | -0.1947 | 0.0213 | 0.4165 | 5.40E-20 | 0.0062 | 0.4165 | 0.0123 | 6.15E-01 |
|  | rs564419538 | 6 | C | G | 0.3801 | 0.0220 | 0.2408 | 5.39E-67 | -0.0426 | 0.2408 | 0.0138 | 2.01E-03 |
|  | rs566368073 | 6 | A | G | 0.9152 | 0.0254 | 0.1081 | 1.00E-200 | 0.0749 | 0.1081 | 0.0517 | 1.47E-01 |
|  | rs568714801 | 6 | G | T | -1.0239 | 0.1106 | 0.0394 | 2.01E-20 | 0.0796 | 0.0394 | 0.0521 | 1.27E-01 |
|  | rs5754104 | 22 | A | G | 0.0891 | 0.0139 | 0.2317 | 1.36E-10 | -0.0086 | 0.2317 | 0.0100 | 3.92E-01 |
|  | rs59117977 | 14 | T | C | -0.1492 | 0.0203 | 0.2589 | 1.78E-13 | -0.0573 | 0.2589 | 0.0355 | 1.07E-01 |
|  | rs59716545 | 17 | G | T | 0.0759 | 0.0120 | 0.5424 | 2.68E-10 | -0.0123 | 0.5424 | 0.0080 | 1.23E-01 |
|  | rs6011186 | 20 | T | C | -0.1074 | 0.0171 | 0.3342 | 3.19E-10 | -0.0252 | 0.3342 | 0.0170 | 1.37E-01 |
|  | rs60726493 | 15 | T | C | 0.0852 | 0.0138 | 0.3238 | 6.67E-10 | 0.0156 | 0.3238 | 0.0084 | 6.42E-02 |
|  | rs62394491 | 6 | T | C | 0.1059 | 0.0168 | 0.5898 | 2.77E-10 | 0.0049 | 0.5898 | 0.0199 | 8.06E-01 |
|  | rs62422878 | 6 | T | C | 0.1037 | 0.0176 | 0.0879 | 3.57E-09 | 0.0121 | 0.0879 | 0.0103 | 2.38E-01 |
|  | rs6589684 | 11 | A | G | -0.1122 | 0.0153 | 0.1186 | 2.29E-13 | -0.0143 | 0.1186 | 0.0099 | 1.47E-01 |
|  | rs660442 | 11 | A | G | -0.1067 | 0.0175 | 0.0936 | 1.11E-09 | -0.0154 | 0.0936 | 0.0099 | 1.19E-01 |
|  | rs6679677 | 1 | A | C | 0.5910 | 0.0230 | 0.0563 | 1.41E-145 | 0.0160 | 0.0563 | 0.0135 | 2.35E-01 |
|  | rs6681482 | 1 | A | G | -0.0903 | 0.0155 | 0.1186 | 6.01E-09 | -0.0055 | 0.1186 | 0.0093 | 5.56E-01 |
|  | rs7097397 | 10 | A | G | -0.0847 | 0.0120 | 0.4000 | 1.42E-12 | -0.0014 | 0.4000 | 0.0088 | 8.73E-01 |
|  | rs71508903 | 10 | T | C | 0.1487 | 0.0143 | 0.1689 | 3.13E-25 | 0.0163 | 0.1689 | 0.0107 | 1.26E-01 |
|  | rs7170107 | 15 | T | C | 0.1366 | 0.0158 | 0.1214 | 6.11E-18 | -0.0097 | 0.1214 | 0.0091 | 2.88E-01 |
|  | rs7206670 | 16 | T | G | 0.0701 | 0.0119 | 0.3852 | 4.14E-09 | 0.0126 | 0.3852 | 0.0086 | 1.41E-01 |
|  | rs7278257 | 21 | C | G | -0.0894 | 0.0143 | 0.4578 | 4.28E-10 | 0.0074 | 0.4578 | 0.0095 | 4.36E-01 |
|  | rs73013527 | 11 | T | C | -0.0803 | 0.0128 | 0.2606 | 3.81E-10 | 0.0061 | 0.2606 | 0.0085 | 4.75E-01 |
|  | rs740122 | 7 | A | G | -0.0782 | 0.0134 | 0.4193 | 5.37E-09 | -0.0194 | 0.4193 | 0.0096 | 4.34E-02 |
|  | rs75187621 | 6 | G | A | -0.1378 | 0.0201 | 0.2325 | 6.41E-12 | -0.0070 | 0.2325 | 0.0342 | 8.38E-01 |
|  | rs75315538 | 6 | G | A | -0.2151 | 0.0390 | 0.1255 | 3.35E-08 | 0.0294 | 0.1255 | 0.0410 | 4.73E-01 |
|  | rs76153210 | 6 | T | C | 0.1597 | 0.0205 | 0.2047 | 6.83E-15 | 0.0017 | 0.2047 | 0.0307 | 9.56E-01 |
|  | rs76739100 | 6 | A | G | 0.3241 | 0.0277 | 0.0414 | 1.60E-31 | 0.0211 | 0.0414 | 0.0260 | 4.16E-01 |
|  | rs7731626 | 5 | A | G | -0.1956 | 0.0184 | 0.1669 | 1.94E-26 | -0.0046 | 0.1669 | 0.0097 | 6.34E-01 |
|  | rs7748243 | 6 | T | C | 0.0674 | 0.0124 | 0.2291 | 4.95E-08 | 0.0085 | 0.2291 | 0.0086 | 3.25E-01 |
|  | rs7749323 | 6 | A | G | 0.2835 | 0.0253 | 0.0461 | 3.47E-29 | -0.0307 | 0.0461 | 0.0248 | 2.17E-01 |
|  | rs7754218 | 6 | T | C | -0.1669 | 0.0156 | 0.5933 | 1.38E-26 | -0.0023 | 0.5933 | 0.0127 | 8.56E-01 |
|  | rs7766988 | 6 | C | G | -0.3317 | 0.0570 | 0.5118 | 5.93E-09 | 0.0603 | 0.5118 | 0.0263 | 2.18E-02 |
|  | rs78434076 | 21 | G | C | 0.1090 | 0.0176 | 0.2531 | 6.24E-10 | -0.0133 | 0.2531 | 0.0281 | 6.36E-01 |
|  | rs78523698 | 6 | C | T | -0.2436 | 0.0265 | 0.1723 | 3.32E-20 | 0.0025 | 0.1723 | 0.0305 | 9.35E-01 |
|  | rs79521532 | 10 | G | A | -0.1938 | 0.0287 | 0.8924 | 1.51E-11 | -0.0130 | 0.8924 | 0.0086 | 1.29E-01 |
|  | rs8032939 | 15 | C | T | 0.1244 | 0.0123 | 0.6126 | 4.47E-24 | 0.0060 | 0.6126 | 0.0093 | 5.18E-01 |
|  | rs8126756 | 21 | C | T | -0.0823 | 0.0137 | 0.5461 | 1.81E-09 | 0.0049 | 0.5461 | 0.0118 | 6.79E-01 |
|  | rs909267 | 6 | C | T | -0.3857 | 0.0230 | 0.3027 | 5.93E-63 | 0.0030 | 0.3027 | 0.0150 | 8.42E-01 |
|  | rs916287 | 6 | C | G | 0.1426 | 0.0229 | 0.9395 | 4.52E-10 | -0.0037 | 0.9395 | 0.0129 | 7.74E-01 |
|  | rs9258206 | 6 | A | G | -0.2167 | 0.0210 | 0.1453 | 5.13E-25 | 0.0288 | 0.1453 | 0.0258 | 2.64E-01 |
|  | rs9271365 | 6 | G | T | 0.4888 | 0.0128 | 0.5857 | 1.00E-200 | 0.0353 | 0.5857 | 0.0081 | 1.18E-05 |
|  | rs9272050 | 6 | A | G | -0.5091 | 0.0144 | 0.3827 | 1.00E-200 | -0.0576 | 0.3827 | 0.0082 | 1.88E-12 |
|  | rs9296059 | 6 | C | A | 0.1518 | 0.0235 | 0.3897 | 1.04E-10 | 0.0385 | 0.3897 | 0.0141 | 6.50E-03 |
|  | rs9405192 | 6 | A | G | -0.0890 | 0.0137 | 0.3995 | 9.26E-11 | 0.0185 | 0.3995 | 0.0094 | 4.79E-02 |
|  | rs947474 | 10 | A | G | 0.0948 | 0.0162 | 0.5802 | 5.24E-09 | 0.0040 | 0.5802 | 0.0105 | 7.04E-01 |
|  | rs9532434 | 13 | C | T | 0.1140 | 0.0126 | 0.7956 | 1.94E-19 | -0.0088 | 0.7956 | 0.0087 | 3.10E-01 |
|  | rs9693589 | 8 | A | G | 0.1127 | 0.0128 | 0.4006 | 1.50E-18 | 0.0157 | 0.4006 | 0.0093 | 9.15E-02 |
|  | rs9784858 | 6 | C | G | 0.2222 | 0.0178 | 0.3530 | 1.00E-35 | 0.0478 | 0.3530 | 0.0132 | 2.97E-04 |
|  | rs9927316 | 16 | G | C | 0.0906 | 0.0136 | 0.2837 | 2.30E-11 | -0.0014 | 0.2837 | 0.0107 | 8.96E-01 |
|  | rs9943599 | 11 | T | C | 0.0830 | 0.0131 | 0.3545 | 2.70E-10 | -0.0233 | 0.3545 | 0.0082 | 4.68E-03 |
|  | rs9979383 | 21 | T | C | 0.0735 | 0.0117 | 0.4087 | 3.17E-10 | 0.0052 | 0.4087 | 0.0090 | 5.64E-01 |
| SLE | rs1131114 | 6 | C | T | 0.4689 | 0.0843 | 0.1826 | 2.71E-08 | -0.0530 | 0.0093 | 0.2456 | 1.21E-08 |
|  | rs41272536 | 1 | G | A | 1.0971 | 0.1855 | 0.0378 | 3.33E-09 | -0.0351 | 0.0216 | 0.0447 | 1.04E-01 |
|  | rs9268178 | 6 | T | C | 0.8917 | 0.1130 | 0.0969 | 3.01E-15 | -0.0518 | 0.0129 | 0.1079 | 5.86E-05 |
|  | rs9274503 | 6 | T | C | 0.4218 | 0.0702 | 0.3540 | 1.87E-09 | -0.0177 | 0.0083 | 0.4420 | 3.20E-02 |
| Hyperthyroidism | rs10087240 | 8 | T | C | 0.0011 | 0.0002 | 0.4571 | 3.30E-09 | 0.0021 | 0.0080 | 0.4690 | 7.93E-01 |
|  | rs112335954 | 6 | A | T | 0.0031 | 0.0003 | 0.2196 | 7.20E-28 | -0.0179 | 0.0310 | 0.0216 | 5.64E-01 |
|  | rs113664977 | 6 | G | C | 0.0037 | 0.0004 | 0.1505 | 1.80E-24 | 0.0266 | 0.0313 | 0.0307 | 3.95E-01 |
|  | rs12741781 | 1 | G | T | 0.0011 | 0.0002 | 0.3269 | 4.30E-09 | 0.0011 | 0.0081 | 0.3291 | 8.92E-01 |
|  | rs13250295 | 8 | T | C | -0.0011 | 0.0002 | 0.3100 | 1.90E-08 | 0.0056 | 0.0092 | 0.2962 | 5.43E-01 |
|  | rs148967059 | 6 | A | T | -0.0025 | 0.0002 | 0.2090 | 1.30E-25 | 0.0325 | 0.0326 | 0.0173 | 3.19E-01 |
|  | rs1559810 | 3 | A | C | 0.0010 | 0.0002 | 0.4072 | 2.90E-08 | -0.0184 | 0.0086 | 0.4210 | 3.30E-02 |
|  | rs1794279 | 6 | T | G | 0.0068 | 0.0003 | 0.1258 | 6.50E-134 | -0.0509 | 0.0130 | 0.1054 | 9.50E-05 |
|  | rs185320691 | 6 | C | G | -0.0023 | 0.0003 | 0.1042 | 1.60E-12 | 0.0136 | 0.0225 | 0.0482 | 5.46E-01 |
|  | rs185774696 | 6 | T | C | 0.0031 | 0.0002 | 0.2967 | 3.20E-40 | 0.0353 | 0.0542 | 0.0092 | 5.15E-01 |
|  | rs2160215 | 14 | C | T | 0.0027 | 0.0002 | 0.3758 | 1.70E-45 | -0.0038 | 0.0083 | 0.3587 | 6.47E-01 |
|  | rs2394186 | 6 | G | A | -0.0016 | 0.0002 | 0.1705 | 3.40E-11 | 0.0208 | 0.0107 | 0.1660 | 5.17E-02 |
|  | rs2523590 | 6 | C | T | -0.0026 | 0.0002 | 0.3177 | 3.10E-40 | 0.0055 | 0.0086 | 0.2960 | 5.23E-01 |
|  | rs28752803 | 6 | T | C | 0.0028 | 0.0003 | 0.3401 | 6.80E-28 | -0.0303 | 0.0087 | 0.5643 | 4.82E-04 |
|  | rs3087243 | 2 | A | G | -0.0018 | 0.0002 | 0.4507 | 7.50E-24 | -0.0018 | 0.0084 | 0.4291 | 8.30E-01 |
|  | rs3129294 | 6 | C | A | -0.0014 | 0.0002 | 0.3471 | 6.70E-13 | -0.0053 | 0.0084 | 0.3439 | 5.26E-01 |
|  | rs409602 | 5 | A | T | 0.0014 | 0.0003 | 0.1446 | 4.60E-08 | -0.0100 | 0.0117 | 0.1410 | 3.91E-01 |
|  | rs4409785 | 11 | C | T | 0.0015 | 0.0002 | 0.1726 | 9.20E-10 | -0.0002 | 0.0090 | 0.1712 | 9.82E-01 |
|  | rs534331448 | 6 | C | T | 0.0016 | 0.0002 | 0.4194 | 6.40E-15 | 0.0280 | 0.0613 | 0.0089 | 6.48E-01 |
|  | rs553276313 | 6 | A | C | 0.0030 | 0.0002 | 0.3513 | 4.30E-45 | 0.0394 | 0.0206 | 0.0641 | 5.63E-02 |
|  | rs6679677 | 1 | A | C | 0.0025 | 0.0003 | 0.1008 | 1.40E-16 | 0.0160 | 0.0135 | 0.1004 | 2.35E-01 |
|  | rs6906566 | 6 | T | C | -0.0023 | 0.0003 | 0.1524 | 2.60E-20 | -0.0075 | 0.0110 | 0.1567 | 4.94E-01 |
|  | rs7090530 | 10 | A | C | 0.0011 | 0.0002 | 0.6021 | 4.50E-09 | 0.0126 | 0.0081 | 0.6067 | 1.22E-01 |
|  | rs71536554 | 6 | C | T | 0.0033 | 0.0003 | 0.1627 | 5.20E-24 | 0.0114 | 0.0188 | 0.0709 | 5.45E-01 |
|  | rs71542456 | 6 | G | A | 0.0042 | 0.0003 | 0.2046 | 2.90E-51 | -0.0400 | 0.0122 | 0.1464 | 1.08E-03 |

## **Supplementary Table 1.** 133 SNPs, 4 SNPs, and 25 SNPs, respectively, were the characteristics of genetic variations linked to RA, SLE, and hyperthyroidism and their effects on PCa.
